# Treatment of Gonorrheal Vulvovaginitis in Young Girls

**Published:** 1912-09

**Authors:** 


					﻿Treatment of Gonorrheal Vulvovaginitis in Young Girls.
—Dr. Tieche of Zurich (Correspondence Blatt, fur Schweizer
Aerzta, No. 5) reports the following, interesting case: “A girl, 2
years of age, was infected through the mother with exudation for
over two months. Treatment commenced December 5, 1910.
Daily injection per urethra and vagina of 10 c.c. of £ per cent
solution of Syrgol. Washed daily with boracic lotion. December
12, 1910, no material change, 1 per cent solution recommended.
December 25 slight watery discharge, no gonococci. Mother’ is
continuing treatment. January 12, 1911, a few leucocytes in mu-
cus, but no discharge. No secretion, no gonococci in slime, Feb-
ruary 20, 1911.
“I have frequently seen a complete cure in from five to eight
weeks, by means of | to 1 per cent nitrate of silver, but relapses
could not be avoided, although they did not occur in the majority
of cases. It has not been demonstrated that relapses will not take
place after Arthigon injections.”
A case of vulvovaginitis which came under the writer’s notice
in 1909 was complicated with rectal gonorrhea in a girl of 7 years.
This was a very intractable case with frequent, painful, sliniy
motions, charged with leucocytes and gonococci. Such complica-
tions are rare, and it is the first case he had met. Kaumheimer
describes a rectal case with these symptoms which were the prom-
inent features in the above case.. It can not be denied that re-
currence may take place from chronic rectal gonorrhea. It is
therefore advisable to examine the stools carefully for the slime
indications. The action of vaccine on rectal gonorrhea is at present
unknown, therefore local treatment with Ichthyol suppositories of
Syrgol cones is the safest course to follow. Good results were
obtained in female gonorrhea by urethral injections of 1 to 2 per
cent solution of Syrgol and douching with 1 to 3 per cent, also
tampons soaked with | per cent solution.
The author, therefore, strongly recommends this preparation of
silver in the treatment of vulvovaginitis.
				

